# Genetic differentiation in the soil-feeding termite *Cubitermes *sp. *affinis subarquatus*: occurrence of cryptic species revealed by nuclear and mitochondrial markers

**DOI:** 10.1186/1471-2148-6-102

**Published:** 2006-11-23

**Authors:** Virginie Roy, Christine Demanche, Alexandre Livet, Myriam Harry

**Affiliations:** 1 UMR 137 Biosol, UFR de Sciences, Université Paris XII – Val de Marne, av. du Général de Gaulle, 94010 Créteil cedex, France; 2 Faculté de Pharmacie, Laboratoire de Parasitologie, 3 rue du Pr. Laguesse, 59006 Lille, France

## Abstract

**Background:**

Soil-feeding termites are particularly interesting models for studying the effects of fragmentation, a natural or anthropic phenomenon described as promoting genetic differentiation. However, studying the link between fragmentation and genetics requires a method for identifying species unambiguously, especially when morphological diagnostic characters are lacking. In humivorous termites, which contribute to the fertility of tropical soils, molecular taxonomy and phylogenetic relationships are rarely studied, though mitochondrial and nuclear molecular markers are widely used in studies of pest termites. Here, we attempt to clarify the taxonomy of soil-feeding colonies collected throughout the naturally fragmented Lopé Reserve area (Gabon) and morphologically affiliated to *Cubitermes *sp. *affinis subarquatus*. The mitochondrial gene of cytochrome oxidase II (COII), the second nuclear rDNA internal transcribed spacer (ITS2) and five microsatellites were analyzed in 19 colonies.

**Results:**

Bayesian Inference, Maximum Likelihood and Maximum Parsimony phylogenetic analyses, which were applied to the COII and ITS2 sequences, and Neighbor-Joining reconstructions, applied to the microsatellite data, reveal four major lineages in the *Cubitermes *sp. *affinis subarquatus *colonies. The concordant genealogical pattern of these unlinked markers strongly supports the existence of four cryptic species. Three are sympatric in the Reserve and are probably able to disperse within a mosaic of forests of variable ages and savannahs. One is limited to a very restricted gallery forest patch located in the North, outside the Reserve.

**Conclusion:**

Our survey highlights the value of combined mitochondrial and nuclear markers for exploring unknown groups such as soil-feeding termites, and their relevance for resolving the taxonomy of organisms with ambiguous morphological diagnostic characters.

## Background

It has been suggested that ecosystem fragmentation has important consequences for animal populations by reducing and dividing the distribution areas and by limiting connections between the fragments [1,2]. As a result, this natural or anthropological phenomenon is described as a process promoting the genetic differentiation of isolated populations and could be involved in speciation. Insects are interesting models for studying the effects of fragmentation owing to their abundance, limited dispersal, short generation time and sensitivity to disturbances. In tropical ecosystems, termites (Isoptera) may represent up to 95% of soil insect biomass; humivorous termites contribute to tropical soil fertilization [3-6]. Since soil-feeders are very sensitive to changes in their environment, they constitute interesting bio-indicators in landscape fragmentation studies, when the evolution of their specific richness is surveyed [7-9].

Studying the link between genetics and fragmentation requires evaluation of breeding structure, gene flow and genetic differentiation among populations. This implies unambiguous identification of species. For a long time, termite systematics was based on morphological and\or morphometrical character sets for individuals belonging to various castes (alates, soldiers or workers). During the past decade, an increasing number of taxonomic studies have shown that molecular methods constitute fast and reliable diagnostic systems, which complement morphological identification. Indeed, several studies based on mitochondrial genome sequences such as the cytochrome oxidase genes, the AT-rich region and the 16S rDNA have thrown a great deal of light on termite taxonomy and on phylogenetic and phylogeographic analyses of the *Reticulitermes *[10-15], *Nasutitermes *[16-18] and *Heterotermes *[19] genera. Because mitochondrial gene trees do not necessarily reflect species trees owing to their maternal inheritance, the addition of nuclear markers in molecular studies is useful for confirming the organismal phylogeny [20]. Among the nuclear sequences, the internal transcribed spacers (ITS) of rDNA are more polymorphic between than within species. It is generally assumed than concerted evolution homogenizes individual rDNA repeats and produces a mostly uniform sequence in all repeats in a given species. Although there is intra-individual variation in some taxa, the ITS2 region sequences are considered phylogenetically informative and able to distinguish closely-related species [21]. Such an evolutionary pattern has allowed sibling or cryptic species in the *Reticulitermes *genus to be discriminated [22-24]. Microsatellites are also very useful nuclear polymorphic markers and have contributed widely to the resolution of colony and population genetic structures in Isoptera [25-27]. Many surveys have concerned the xylophageous genus,*Reticulitermes*, in non-natural fragmented areas such as urban ecosystems [28,29]. To date, however, microsatellite markers have not been used to investigate termite phylogeny or species discrimination, as has been done for other groups *e.g*. vertebrates [30], ticks [31], wasps [32] and ants [33].

In the present survey, we studied the humivorous afro-tropical *Cubitermes *genus (Termitidae, Termitinae), which shows remarkable ecological plasticity, colonizing savannahs or forests according to species. Among the soil-feeding Termitidae, the *Cubitermes *genus is one of the main builders; its nest can shelter up to 10 genera of true inquiline or optional builder termites [34]. In spite of its undeniable ecological interest, the taxonomy of this genus is poorly resolved and a complete revision appears necessary [35]. Indeed, at least 64 species of *Cubitermes *have been described on the basis of morphometrical characters, but it is strongly suspected that some of these are synonymic. It is important to note that extensive taxonomic work is necessary for the West African species since the sole currently available key concerns the East African species [36]. Molecular data are also lacking for the *Cubitermes *genus, as only one sequence from cytochrome oxidase I [37] and two from 12S mtDNA [GenBank: AF475037, AF475001] are registered.

The aim of this work is to clarify the taxonomic status of the *Cubitermes *colonies from the Lopé Reserve region (Gabon), in order to obtain a better understanding of tropical termite diversity in fragmented areas. The Lopé Reserve is typically characterized by a mosaic of forests and savannahs and constitutes an ideal setting for studies of natural fragmentation.

**Figure 1:**
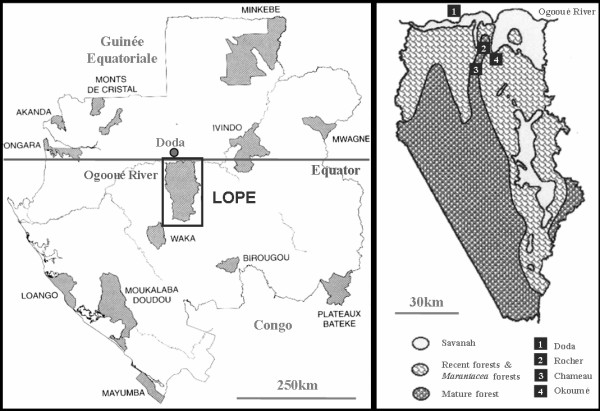
**Map of the study area**. A. Map of the National Parks of Gabon since 2002, modified from [58]: location of the Lopé Reserve. B. Map of the Lopé Reserve region, modified from [59]: location of the four sites: Doda, Rocher, Chameau and Okoumé and landscape types.

On the basis of morphological comparisons with type specimens in the collections of the British Museum (London) and the Royal Museum for Central Africa (Tervuren), and because they showed no diagnostic morphometrical variation or molecular divergence in mitochondrial 12S and 16S rDNA (Harry, unpublished data), the *Cubitermes *colonies were affiliated to a single species, *Cubitermes *sp. *affinis subarquatus *(Sjöstedt). Here we attempt to reconstruct a phylogeny based on three types of polymorphic and independent molecular markers. We sequenced a portion of the mitochondrial COII gene and the ITS2 region, and determined the genotypes at five microsatellite loci isolated from *Cubitermes subarquatus *[38], for 19 *Cubitermes *colonies from four different sites (Figure 1). Three of these sites were in the same geographical scale within the Lopé Reserve and corresponded to forest patches of different ages, including small savannah patches: Okoumé (75 years old), Rocher (800 years old) and Chameau (800 years old). A fourth site, Doda, was an isolated gallery-forest outside the Reserve within a savannah landscape.

## Results

### Mitochondrial sequence analyses

Mitochondrial sequence dataset consisted in 558 bp sequenced for one individual from each of the 19 colonies collected in the four sites, and for the outgroup taxa, *Apilitermes longiceps *and *Crenetermes albotarsalis *(Termitidae, Termitinae).

**Figure 2:**
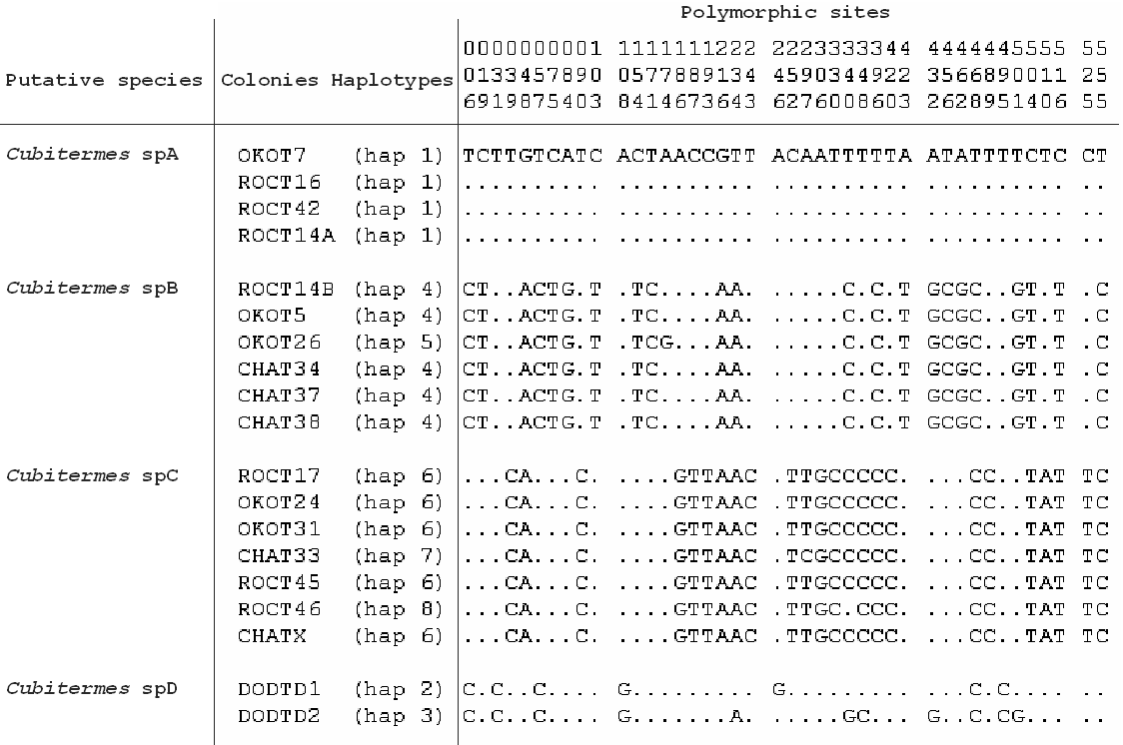
**Polymorphic sites for COII haplotypes**. Haplotypes for each colony (OKO: Okoumé, ROC: Rocher, CHA: Chameau, DOD: Doda) and position of the polymorphic sites in the COII *Cubitermes *sequences.

**Figure 3:**
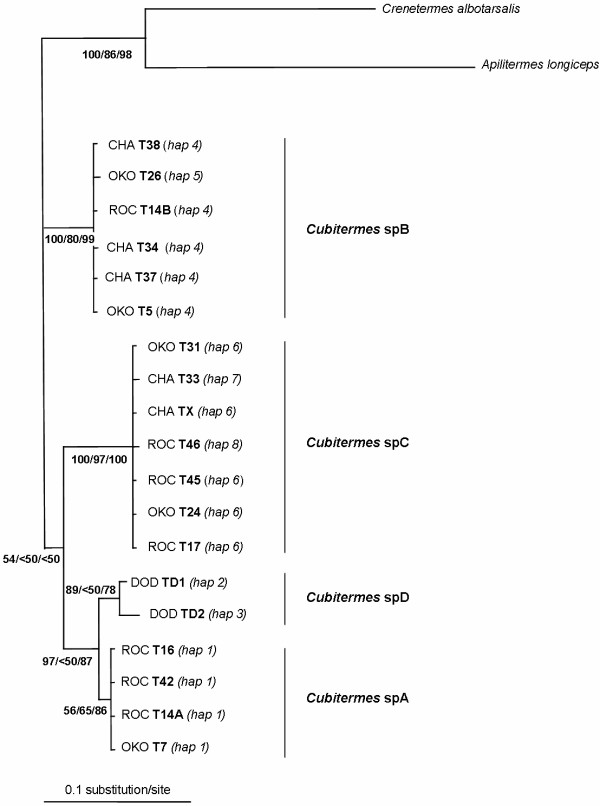
**Bayesian phylogenetic tree of mtDNA haplotypes**. Bayesian tree showing relationships of COII sequences from *Cubitermes *sp. *affinis subarquatus *colonies. *Apilitermes *and *Crenetermes *sequences are used to root the tree. BI posterior probabilities followed by ML and MP bootstrap support values are indicated in bold at nodes. Putative species are indicated in bold on the right.

In total for the *Cubitermes *sequences, 42 nucleotidic sites were variable (7.5%) and the overall proportion of A+T reached 66.7%. Height haplotypes were scored (Table 1 and Figure 2) differing at 1–30 nucleotide sites (0.18–5.40% sequence divergence). Maximum Parsimony (MP), Bayesian Inference (BI) and Maximum Likelihood (ML) reconstructions clearly showed four distinct COII haplotype groups (Figure 3). We named the first clade *Cubitermes *spA, including sequences from the T7, T16, T42 and T14A colonies. The second clade, named *Cubitermes *spB, included T5, T26, T34, T37, T38 and T14B sequences. The third clade, *Cubitermes *spC, comprised T17, T24, T31, T33, T46, T45 and TX sequences and the fourth group, *Cubitermes *spD, only the two sequences from the colonies of the Doda site, TD1 and TD2.

**Table 1:** *Cubitermes *samples and summary of sequence and microsatellite data used to distinguish cryptic species.

**Putative species**	**Colonies**	**Sites**	**COII sequences (N = 1)**		**ITS2 sequences (N = 1)**			**Microsatellite alleles**				
			
			**AN**	**Hap**	**AN**	**Hap**	**N**	**AR P14 (n)**	**AR P19 (n)**	**AR P32 (n)**	**AR P34 (n)**	**AR P41 (n)**
*Cubitermes *spA	OKOT7	Okoumé	DQ127300	1	DQ246528	a	26	227–253 (10)	408–412 (2)	160–174 (2)	231–239 (4)	149 (1)
	ROCT16	Rocher	DQ127302	1	DQ246527	a	24					
	ROCT42	Rocher	DQ127299	1	DQ246529	a	30					
	ROCT14A	Rocher	DQ246540	1	DQ246530	a	6					

*Cubitermes *spB	ROCT14B	Rocher	DQ246542	4	DQ246524	b	12	231–233 (2)	392–408 (4)	160–176 (3)	231–247 (8)	149–155 (3)
	OKOT5	Okoumé	DQ127311	4	DQ246519	b	18					
	OKOT26	Okoumé	DQ127312	5	DQ246520	b	24					
	CHAT34	Chameau	DQ127309	4	DQ246523	b	26					
	CHAT37	Chameau	DQ127310	4	DQ246521	b	48					
	CHAT38	Chameau	DQ127308	4	DQ246522	b	33					

*Cubitermes *spC	ROCT17	Rocher	DQ127304	6	DQ246537	c	23	225–245 (8)	404–408 (2)	160–180 (4)	231–245 (5)	149 (1)
	OKOT24	Okoumé	DQ127303	6	DQ246531	c	19					
	OKOT31	Okoumé	DQ246543	6	DQ246533	c	24					
	CHAT33	Chameau	DQ127307	7	DQ246534	c	19					
	ROCT45	Rocher	DQ246544	6	DQ246535	c	20					
	ROCT46	Rocher	DQ127306	8	DQ246536	c	34					
	CHATX	Chameau	DQ127305	6	DQ246532	c	18					

*Cubitermes *spD	DODTD1	Doda	DQ127301	2	DQ246525	d	24	229–245 (4)	412 (1)	160–178 (2)	233–239 (4)	149 (1)
	DODTD2	Doda	DQ246541	3	DQ246526	d	19					

Outgroups	*A. longiceps*	-	DQ246545	-	DQ246538	-	-	-	-	-	-	-
	*C. albotarsalis*	-	DQ246546	-	DQ246539	-	-	-	-	-	-	-

GenBank accession numbers (AN) and haplotype names (Hap) for COII and ITS2 sequences and allele ranges (AR) for the five microsatellite loci. N, number of individuals tested for each of the 19 *Cubitermes *colonies of the four geographical sites and for *A. longiceps *and *C. albotarsalis *and n, number of microsatellite alleles found in each locus and putative cryptic species.

Sequence divergence was quite low within these groups. Indeed, in the *Cubitermes *spA group, only one haplotype was detected and sequence divergence was inferior to 1% within *Cubitermes *spB and *Cubitermes *spC groups (sequences differed by 0–0.18% and 0–0.36% respectively).

On the opposite, the divergence between the four mitochondrial lineages was high. The *Cubitermes *spA and *Cubitermes *spB sequences differed from each other by 3.94% (net sequence divergence), *Cubitermes *spA and *Cubitermes *spC by 4.22% and *Cubitermes *spB and *Cubitermes *spC by 4.99%. Finally, *Cubitermes *spD diverged by 1.07% from *Cubitermes *spA.

### ITS2 sequence analyses

**Figure 4:**
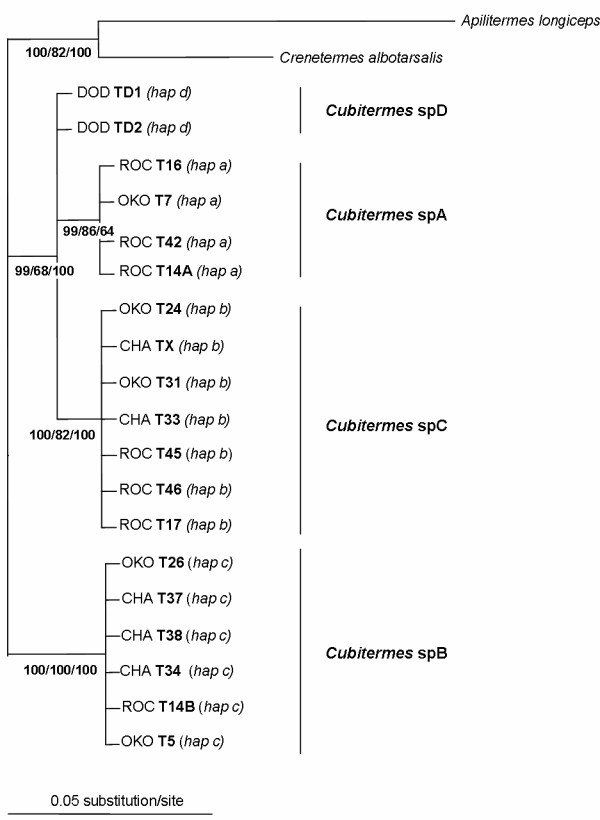
**Bayesian phylogenetic tree of ITS2 sequences**. Bayesian tree showing relationships of ITS2 sequences from *Cubitermes *sp. *affinis subarquatus *colonies. *Apilitermes *and *Crenetermes *sequences are used to root the tree. BI posterior probabilities followed by ML and MP bootstrap support values are indicated in bold at nodes. Putative species are indicated in bold on the right.

A total of 298 positions sequenced for one individual from each of the 19 *Cubitermes *colonies, for *Apilitermes longiceps *and *Crenetermes albotarsalis *were aligned for ITS2 sequences. Regarding *Cubitermes *sequences, we found 262 invariable sites, 25 alignment gaps and 11 polymorphic sites. MP, BI and ML reconstructions confirmed the four lineages found with the COII gene (Figure 4). However, the position of *Cubitermes *spD was quite different since it was not found univocally clustered with *Cubitermes *spA. In this ITS2 tree, high BI posterior probabilities, ML and MP bootstrap support values were found for grouping *Cubitermes *spA, *C*. spD and *C*. spC in the same clade (Figure 4, 99/68/100) whereas a lower resolution of this branch appeared in the tree of mtDNA haplotypes (Figure 3, 54/<50/<50).

Sequence data from ITS2 revealed no polymorphism within the putative four species. Since no heterozygote was detected, ITS2 sequences were assigned to four unique haplotypes (*a *for *Cubitermes *spA, *b *for *Cubitermes *spB, *c *for *Cubitermes *spC, and *d *for *Cubitermes *spD, Table 1).

Again, divergence between groups was high in comparison with within-group divergence. Haplotypes of *Cubitermes *spA differed from the *Cubitermes *spC haplotypes by 2.18% (net sequence divergence). The *Cubitermes *spA and *Cubitermes *spB haplotypes differed from each other by 7.61% and *Cubitermes *spB and *Cubitermes *spC by 8.36%. Finally, the haplotype of *Cubitermes *spD differed from the haplotype of *Cubitermes *spA by 1.43%.

### Microsatellite analyses

A total of 447 individuals for the 19 nests were surveyed at five microsatellite loci. The genetic differentiation among colonies within each putative species (F_CT _= 0.275, 0.258, 0.322 for *Cubitermes *spA, *C*. spB and *C*. spC, respectively) was substantially low. Otherwise, very high genetic differentiation was detected between colonies of different *Cubitermes *putative species. *Cubitermes *spA and *Cubitermes *spD were the less differentiated (F_ST*AD *_= 0.12; CI = -0.09–0.38) followed by *Cubitermes *spA and *Cubitermes *spC groups (F_ST*AC *_= 0.25, CI = 0.11–0.39). *Cubitermes *spA and *Cubitermes *spB (F_ST*AB *_= 0.49, CI = 0.27–0.67) and *Cubitermes *spB and *Cubitermes *spC (F_ST*BC *_= 0.48, CI = 0.25–0.68) showed similar high patterns of differentiation.

**Figure 5:**
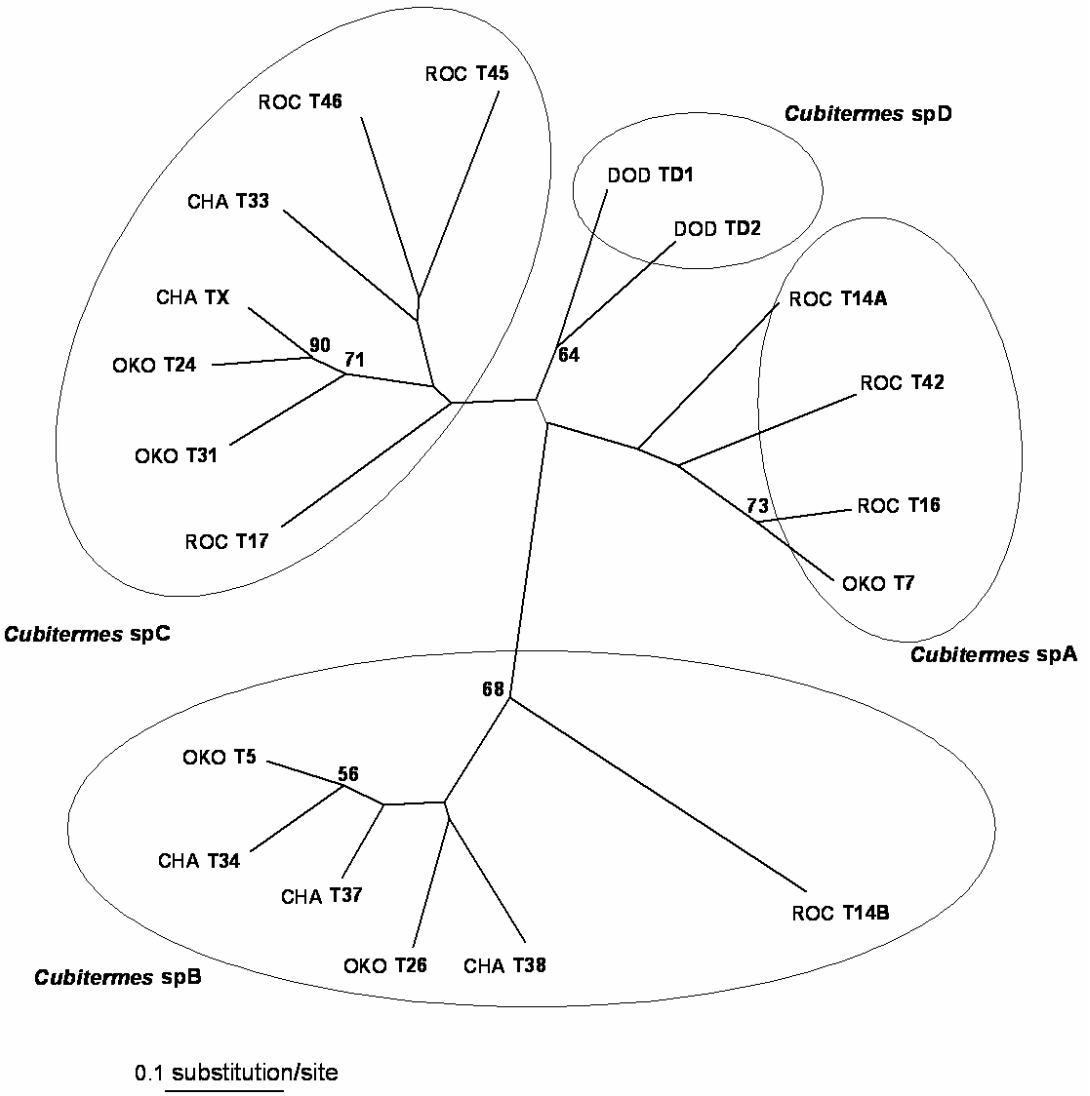
**Neighbor-Joining tree calculated from the microsatellite data**. Distances between *Cubitermes *sp. *affinis subarquatus *colonies are calculated based on the chord distance of Cavalli-Sforza from five microsatellite loci. The tree is unrooted. Values at nodes represent bootstrap support values (only values >50% are shown). Putative species are indicated in bold.

The NJ trees based on the DAS distance, the minimum genetic distance of Nei and the chord distance (D_C_) showed a genetic structure with four main clusters (Figure 5).

## Discussion

In tropical soil-feeding termites, it is impractical to apply a Biological Species Concept [39] because of the difficulties in realizing experimental crosses and observing natural hybridizations. Thus, our purpose here was to apply a Phylogenetic Species Recognition (PSR) based on the Genealogical Concordance Concept [40] to diagnose the *Cubitermes *species or to detect cryptic species. Such a concept has often been applied in bacteria, fungi and animals. In termites, research involving DNA-based taxonomy of structural and agricultural pest species is important [10]. Comparison of multiple molecular markers has allowed the taxonomic status of new species in *Reticulitermes *to be assessed [60,61] and has provided evidence for species synonymy in this genus [12,14,23]. Many DNA-based species recognitions are corroborated by evidence of morphological/chemical differences or geographically defined groups. However, PSR can be a powerful tool for diagnosing otherwise undistinguishable species, because genetic changes occurring in recently-isolated species may be observed before morphological or behavioral changes arise [41]. As found recently in other social insects, genetic isolation is not always accompanied by evident morphological differentiation. In fire ants belonging to the *Solenopsis *genus, genetic analyses based on allozyme and mitochondrial markers demonstrated the occurrence of sympatric and indistinguishable cryptic species [42-44].

The genetic results obtained in our study from the combined mitochondrial, nuclear and microsatellite markers unequivocally show deep separation among four groups of genotypes in the *Cubitermes *sp. *affinis subarquatus *colonies from the Lopé Reserve region. The congruence of these three types of unlinked molecular markers strongly supports the existence of differentiated genetic pools in this limited area. The mitochondrial and nuclear sequences show very little variation within the four groups but there is considerable variation among them. Although the allele ranges overlap for all the loci, possibly because of size homoplasy, the microsatellite data confirm the occurrence of four differentiated groups. These unexpected levels of subdivision are unlikely to have arisen under gene flow, so these four newly-detected groups can be seen as good evidence for the existence of cryptic species previously unrecognized by morphological techniques.

The lack of detectable morphological differences among *Cubitermes *species is not surprising in view of the data in the taxonomic literature, which imply that the genus is highly complex (great number of species, synonymy, missing data, etc.) [35]. Furthermore, the boundaries between nominal *Cubitermes *species are often concealed by intra-specific morphometrical variability. In his revision of the East African *Cubitermes *species, Williams [36] mentioned that most of the specific characters vary greatly in size and can also vary markedly in shape and proportion within a particular species.

In addition, our genetic data inform the current debate about the use of comparative phylogenetic methods for studying present-day species distributions. These distributions do not necessarily reflect the geographical range of the ancestral species at the time of speciation, because geographical distributions are often labile owing to climate fluctuations, territory expansion or extinction of competitors. In particular, we have certainly not sampled the entire species range and enabled definite conclusions to be drawn about biogeographic differentiation and speciation. However, the repartition of the cryptic *Cubitermes *species is quite interesting in relation to the species distributions of poorly dispersive insects.

In our study, we can consider two landscape units: (1) the Lopé Reserve south of the Ogooué River, where *Cubitermes *spA, *C*. spB and *C*. spC live sympatrically in the closely apposed sites of Okoumé, Chameau and Rocher, the only exception being that *Cubitermes *spA is absent from the Chameau site; (2) the Doda zone to the north, where *Cubitermes *spD is restricted to the distant and isolated gallery-forest of Doda and represents the only *Cubitermes *species in the Doda zone across 10 km. The ecological differentiation of the two zones results from paleogeographic events. Indeed, since the last glaciations, the North Ogooué has progressively run dry, resulting in the regression of all forest types except riparian. In the South Ogooué, a wet environment has been maintained by the well-developed hydro-geographical network and this has allowed the forest to be preserved.

The contemporary sympatric distributions of three of the *Cubitermes *species (*Cubitermes *spA, *C*. spB and *C*. spC) in the Reserve zone could reflect their ability to disperse within a mosaic of forests of variable ages (*e.g*. differing in biotic and edaphic parameters) and savannah "buffer-zones". Indeed, their dispersion seems not to be affected by fragmentation on this small scale. Little is known about the dispersion modalities and the reproductive strategies of the *Cubitermes *species. It is very likely that swarming (*i.e*. alate dispersal flight) is the main mode of dispersal in this genus, since budding (*i.e*. local secondary reproduction initiated by the differentiation of neotenic reproductives, derived from the nymphs or workers of the colony) is not as common in Termitidae as in lower termites [45]. It has been suggested that although the active flight of winged termites is very limited (a few hundreds of meters), the meteorological conditions accompanying dispersal flights could strongly influence the distance covered by these sexual alates. Actually, a recent genetic study of *Macrotermes michaelseni *(Termitidae) suggested that some winged termites can travel considerable distances (50 km), most likely by passive drift [46]. Furthermore, the effectiveness of dispersal obviously depends upon the number of alates and the rate of predation [47].

Finally, the particular distribution pattern of *Cubitermes *spD raises the question of the link between the history of the forest fragmentation and the modalities of speciation. One can indeed wonder whether the history of successive modifications of habitats in this geographical zone has not contributed to the isolation of the termite species populating it. Areas such as isolated gallery forests, like Doda, may constitute refuges for fauna, where some species are led to disappear while others begin to differentiate under the influence of genetic drift.

## Conclusion

The combination of mitochondrial and nuclear markers provides a reliable diagnostic method for separating *Cubitermes *species and offers a complement to the morphometrical diagnostic. Furthermore, these molecular markers could reveal useful information about their phylogenetical relationships. Similarly, such methods could be extended to termites for which species taxonomy is ambiguous.

## Methods

### Study site and species

The field collections were carried out in the Lopé Reserve region (Middle Ogooué, Gabon). The Lopé Reserve is constituted by a mosaic of forest and savannah, primarily formed during the last glaciations (-18000, -12000) and then maintained by human savannah burnings, resulting in a fragmented landscape. The sample area was composed of three forest sites in the Reserve, characterized by vegetation age and 2–5 km apart (Okoumé, Chameau and Rocher). The Okoumé site consisted of 75 years-old *Milletia*, *Aucoumea *and *Marantacea *stands. The Rocher and Chameau sites were older (800 years-old) and consisted in mature forest scattered by rock outcrops and classical pluvial forest stands, respectively. Finally, a fourth sampled site, Doda, was an isolated gallery-forest in a savannah landscape, dating up to 2500 years and situated outside the Reserve. In total, 19 colonies of *Cubitermes *sp. *affinis subarquatus *and two colonies of *Apilitermes longiceps *and *Crenetermes albotarsalis *were sampled (Table 1). Immediately following collection, individuals were placed in absolute ethanol and stored at 4°C in the laboratory until DNA extraction.

### DNA extraction

Total genomic DNA from *Cubitermes*, *Apilitermes *and *Crenetermes *individuals was isolated using an extraction method with *Wilson *buffer (Tris hydrogen chloride 1 M, ethylenediaminetetraacetic acid 0.5 M, sodium chloride 4.5 M, sodium dodecylsulfate 20%, dithiothreitol, proteinase K) followed by a salting-out procedure.

### Mitochondrial and ITS2 sequence analyses

DNA analyses were based on sequences from partial mitochondrial cytochrome oxidase subunit II gene (COII) and nuclear internal transcribed spacer 2 (ITS2). PCR was performed for one individual per colony (N = 19 *Cubitermes *+ 1 *Apilitermes *+ 1 *Crenetermes*) in a total volume of 40 μL (50 μL for ITS2), composed of 20 μL (25 μL) of Taq PCR Master Mix (Qiagen), 1.6 μL (4 μL) of each primer (10 pM), 15.2 μL (18.5 μL) of distilled water and 1.6 μL (2.5 μL) of template DNA. Primers for COII amplification were forward *modified A-tLeu *5'-CAGATAAGTGCATTGGATTT-3' and reverse *B-tLys *5'-GTTTAAGAGACCAGTACTTG-3' [48,49], modified by Miura *et al*. [16]. ITS2 sequences were amplified using forward *ITS2F *5'-TGTGAACTGCAGGACACAT-3' and reverse *ITS2Rcub *5'-ATTCGGCGGGTAGTCTCG-3' primers modified in this study from Jenkins *et al*. [23]. The amplification conditions were adapted from Miura *et al*. [16]. The amplification products were purified using a DNA and Gel band Purification kit (GFX TM PCR kit, Amersham Biosciences USA). Sequence reactions were performed using BigDye Terminator Cycle Sequencing kit version 1.1 (Applied Biosystems), then purified with an ethanol-Na acetate method. Sequence data were obtained using an automatic DNA sequencer (Applied Biosystems, ABI PRISM 310) and analysed with Sequencing Data software (Applied Biosystems). All sequences were registered in GenBank database with accession numbers listed in Table 1.

COII and ITS2 sequences were aligned using CLUSTALW with the default settings [50] and sequences from *Apilitermes longiceps *and *Crenetermes albotarsalis *(Termitidae, Termitinae) were added to both datasets in order to root the trees. Phylogenetic analyses were performed using Maximum Parsimony (MP), Bayesian Inference (BI) and Maximum Likelihood (ML) methods.

MP trees were constructed using Phylip (Phylogeny Inference Package) Version 3.572 [51] with the SEQBOOT, DNAPARS and CONSENSE programs with 1000 repetitions of bootstrap.

For probabilistic methods, a nucleotide substitution model was selected for each sequence dataset using the Akaike Information Criterion (AIC) implemented in Modeltest v3.7 [52]. The best-fit substitution model selected was TrN+G (Nst = 6, Rates = gamma, Pinvar = 0) for COII sequences and TVM (Nst = 6, Rates = equal, Pinvar = 0) for ITS2 sequences. BI trees were constructed using MrBayes 3.1 [53]. We ran four Markov chains (one cold, three heated) for 1,000,000 generations, sampled every 100 generations (burnin = 2500, according to the convergence diagnostic). Consensus trees were generated including posterior probability of clades and branch lengths. ML trees were constructed using thePAUP 4.0b10 program [54] and the reliability of the inferred trees was tested by 100 bootstrap resamplings for COII and 40 for ITS2. Tree topologies were congruent across all methods and therefore, only BI trees are reported with posterior probabilities and bootstrap support values for ML and MP.

### Microsatellite analyses

The genotypes for 18 to 48 sterile individuals from each colony except two (T14A, n = 6 and T14B, n = 12) *i.e*. a total of 447 individuals genotyped, were assayed at five microsatellite loci (P14, P19, P32, P34 and P41) by means of Polymerase Chain Reaction (PCR) amplification. Primer sequences and amplification conditions are given in Harry *et al*. [38]. PCR products were electrophoresed on an ABI Prism 310 DNA sequencer (Applied Biosystems) and microsatellite allele sizes were scored using the GENSCAN and GENOTYPER programs (Applied Biosystems).

We investigated whether genetic differentiation occurs among groups detected in the phylogenetic analyses. In order to do this, we estimated F_ST _coefficients with individuals nested in colonies and colonies nested in major lineages using a three-level hierarchy in the GDA program [55], for each pair of putative cryptic species. 95% CIs were constructed by bootstrapping over loci with 1000 replications. Values for which 95% CIs did not overlap zero were considered as significantly different at the 0.05 level. We compared the allele differentiation among colonies of the same genetic group and among colonies of the same geographical site using the F_CT _coefficient [56] calculated with a two-level hierarchy in the GDA program.

Phylogenetic distances between colonies were estimated with the Populations 1.2.00 program [57] by using the genetic distance of shared alleles, the minimum genetic distance of Nei and the chord distance of Cavalli-Sforza. The resulting genetic distances and bootstrapping procedures (1000 replicates) were used to construct an unrooted consensus tree. Tree topologies were congruent across the three methods and therefore, only one reconstruction is presented here (reconstruction based on the chord distance of Cavalli-Sforza).

## Authors' contributions

VR and CD carried out the molecular genetic studies, participated at the data analysis and the manuscript draft. AL intensively helped VR and CD in DNA sequencing. MH initiated the study, realized the sampling, contributed to the analysis of the results and to the writing of the paper. All authors read and approved the final manuscript.
